# Concentrations-dependent effect of exogenous abscisic acid on photosynthesis, growth and phenolic content of *Dracocephalum moldavica* L. under drought stress

**DOI:** 10.1007/s00425-021-03648-7

**Published:** 2021-05-25

**Authors:** Vahideh Khaleghnezhad, Ali Reza Yousefi, Afshin Tavakoli, Bahman Farajmand, Andrea Mastinu

**Affiliations:** 1 Department of Plant Production & Genetics, University of Zanjan, Zanjan, Iran; 2 Department of Chemistry, College of Science, University of Zanjan, Zanjan, Iran; 3 Department of Molecular and Translational Medicine, University of Brescia, 25123 Brescia, Italy

**Keywords:** Biological yield, Leaf area, Leaf dry weight, Phenolic content, Seed yield

## Abstract

**Main Conclusion:**

The drought conditions and the application of ABA reduce the photosynthetic activity, and the processes related to the transpiration of *Dracocephalum moldavica* L. At the same time, the plant increases the production of phenolic compounds and essential oil as a response to stress conditions.

**Abstract:**

In the semi-arid regions, drought stress is the most important environmental limitations for crop production. Abscisic acid (ABA) plays a crucial role in the reactions of plants towards environmental stress such as drought. Field experiments for two consecutive years in 2016 and 2017 were conducted to evaluate the effect of three watering regimes (well-watered, moderate and severe drought) and five exogenous ABA concentrations (0, 5, 10, 20 and 40 μM) on growth, photosynthesis, total phenolic and essential oil content of *Dracocephalum moldavica* L. Without ABA application, the highest photosynthetic rate (6.1 μmol CO_2_ m^−2^ s^−1^) was obtained under well-watered condition and, moderate and severe drought stress decreased photosynthesis rate by 26.39% and 34.43%, respectively. Some growth parameters such as stem height, leaf area, leaf dry weight and biological yield were also reduced by drought stress. ABA application showed a decreasing trend in photosynthesis rate and mentioned plant growth parameters under all moisture regimes. The highest seed yield (1243.56 kg ha^−1^) was obtained under well-watered condition without ABA application. Increasing ABA concentration decreased seed yield in all moisture regimes. The highest total phenolic content (8.9 mg g^−1^ FW) and essential oil yield (20.58 kg ha^−1^) were obtained from 20 and 5 μM ABA concentration, respectively, under moderate drought stress.

## Introduction

Drought stress is a major constraint to be achieved higher yields in crop plants, especially in the arid and semi-arid regions of the world (Kumar et al. 2020; Mahdavi et al. 2020; Naservafaei et al. 2021; Rad et al. 2020; Reza Yousefi et al. 2020). The intensity and frequency of drought are likely to increase as a result of the predicted future climate change (Wassmann et al. 2009; Aghajanlou et al. 2021). The global average of yield decrease due to drought projected to be more than 50% (Wang et al. 2003). Moisture deficiency induces various physiological and metabolic responses like stomatal closure limiting decline in growth and photosynthesis rate (Flexas and Medrano 2002). In addition, both cell expansion and cell division, two primary processes involved in plant growth, can be influenced by relatively mild drought stress, even before photosynthesis or respiration is affected critical processes, such as germination, emergence, leaf expansion, root and shoot development, dry matter accumulation, floral initiation, pollination, fertilization, seed growth and seed yield (Morgan 1984; Roberts 1988). Among them, leaf expansion is the most sensitive processes (Alves and Setter 2004). Indeed, drought decrease cell size and cell number and thus can lead to decreased leaf area, stem growth and plant height (Randall and Sinclair 1988; Simonneau et al. 1993; Tardieu et al. 2000). In addition, Baher et al. (2002) showed that moderate drought can decrease the length of time from floral initiation (Cruz and O'Toole 1984). Colom and Vazzana (2002) reported that drought reduced total fresh and dry weight of *Satureja hortensis*. Similar results showed that the number of stem per plant and total dry weight was negatively related to water stress in *Eragrostis curvula*. Drought stress during flower development decrease seed numbers (Wheeler et al. 2000; Prasad et al. 2006). In addition, drought decreases the seed-filling duration, leading to smaller seed size and seed yield (Frederick et al. 1991; De Souza et al. 1997; Wardlaw and Willenbrink 2000).

Another significant response of plants to water stress is the increase in the synthesis of secondary metabolites (Lazzari et al. 2012; Cordoba et al. 2021; Eriksen et al. 2021; Jogawat et al. 2021). With the increase in drought, the plant produces different antioxidant molecules such as polyphenols and carotenoids with the aim of counteracting oxidative stress (Jogawat et al. 2021). Humanity has exploited this potential to produce new supplements and pharmaceutical products with antioxidant activity (Lazzari et al. 2012; Mastinu et al. 2012, 2021; Kumar et al. 2019; Gupta et al. 2020).

*Dracocephalum moldavica* L. (*D. moldavica*) belonging to the Lamiaceae (Labiatae) family is a perennial herb. It is native to central Asia and is naturalized in eastern and central Europe. Flowers and all vegetative parts of *D. moldavica* (young leaves and stems) are used due to their aromatic compounds (Amin 1991).The essential oil of *D. moldavica* includes a group of phenolic compounds that belong to terpenoids and phenylpropanoids. These compounds have a critical role in plant defense system and these are used in medicinal and food industries (Charles and Simon 1990). The chemical composition of essential oils varies depend on genetics and environmental conditions (Eyres et al. 2005). Plants produce higher quantities of secondary metabolites under drought conditions (Sangwan et al. 2001). Bettaieb et al. (2011) reported that drought stress on increasing essential oil and total phenolic contents in the aerial parts of cumin (*Cuminum cyminum* L.).

Endogenous abscisic acid (ABA), together with other hormonal signals, regulates the processes of transpiration and foliar photosynthesis, induces stomatal closure, inhibits the growth of the aerial part, regulates seed dormancy, is involved in the response of plants to stress (Moreira et al. 2020). In particular, it plays a vital role in the adaptive growth responses to drought (Lu et al. 2009). Some physiological and morphological responses such as photosynthesis and leaf expansion have been reported to be affected by ABA (Umezawa 2011). Moreover, endogenous ABA can be involved in the biosynthetic pathway of phenolic compounds (Castellarin et al. 2012).

On the other hand, exogenous ABA can act on the synthesis of enzymes involved in the primary and secondary metabolism of the plant and can induce an increase in the synthesis of phenolic compounds such as flavonoids and carotenoids (Stanley and Yuan 2019; Gai et al. 2020). Also, several authors reported an increase in the production of phenolic compounds and others secondary metabolites after exogenous application of ABA (Sandhu et al. 2011; Ferrara et al. 2013; Koyama et al. 2014; Flores et al. 2018; Ghassemi-Golezani et al. 2018). Furthermore, it appears that ABA may be involved in regulating the biosynthesis of plant phenolic compounds in plants subjected to drought stress (González‐Villagra et al. 2018; Gai et al. 2020). However, its application does not always generate univocal and immediate answers in all plant species (Negin and Moshelion 2016).

Even though several strategies have been proposed to mitigate the negative effects of drought stress in plants, to our knowledge there is little information about the physiological role of ABA in drought stress alleviation in *D. moldavica* and no such study has been conducted under field conditions. Therefore, this study was initiated to evaluate the drought tolerance ability of *D. moldavica* and determined the effects of different concentration of exogenous ABA on growth and yield under drought condition. Furthermore, given the involvement of ABA in the synthesis of carotenoids and flavonoids in drought conditions, the content of phenolic compounds in *D. moldavica* was evaluated under water stress conditions.

## Materials and methods

### Plant material and experimental site

**Fig. 1:**
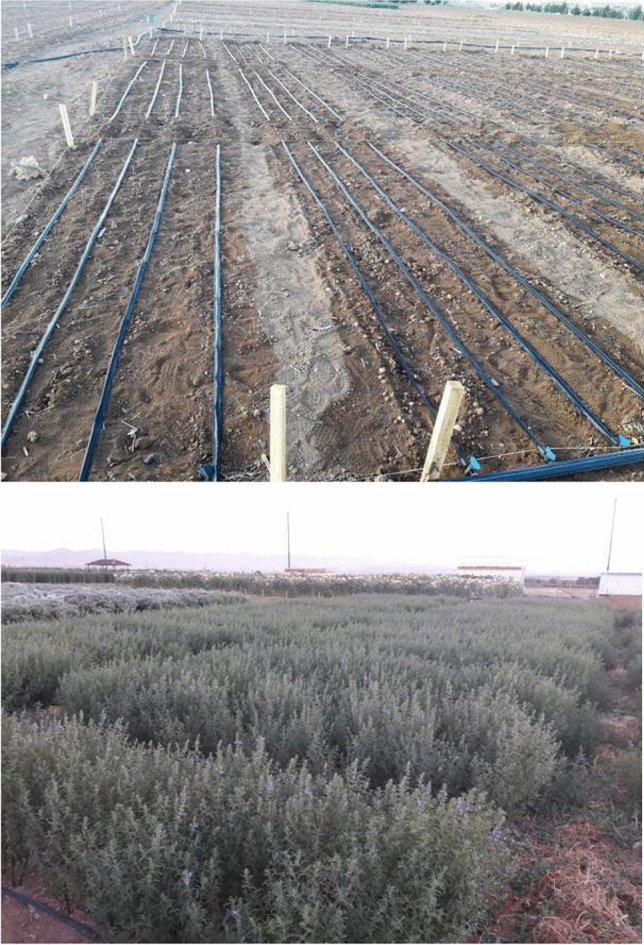
Field crops of *Dracocephalum moldavica* L

Two field experiments were conducted under furrow irrigation at the experimental farm of the University of Zanjan, Zanjan, Iran (36° 41′ N and 48° 29′ E, altitude 1663 m) during the growing seasons of 2016 and 2017 (Fig. 1). The 30-year annual mean temperature and precipitation were 11 ºC and 293 mm, respectively. The region is characterized by a cool semi-arid climate. The soil type was a sandy loam with a pH of 7.32. Soil properties of experimental site including electrical conductivity, organic matter content, total nitrogen, available phosphorus and available potassium were 1.2 (dS/m), 1.75%, 0.2%, 8.4 ppm and 156 ppm, respectively. The land was prepared by plowing and leveling before planting. The seeds of *Dracocephalum moldavica* L. (*D. moldavica*) were obtained from Pakan Seed Company (Isfahan, Iran). *D. moldavica* was sown at 30 seeds m^–2^, at 0.30 m row spacing, on May 12, 2016 and May 11, 2017. Plots were four rows 1.5 m wide by 4 m long.

### Experimental design and treatments

The experiment was arranged in a split-plot based on randomized complete block design with three replications. Three watering regimes [well-watered, moderate drought (delay irrigation up to − 8 bar) and severe drought (delay irrigation up to − 15 bar)] were set as the main plot and different ABA concentrations (0, 5, 10, 20 and 40 µM) were set as subplots. The mentioned water potential to apply drought was determined by the field moisture curve.

The soil water content was monitored daily and, the data were converted to the soil matric suction using soil water characteristics curve (SWC) obtained experimentally. The SWC was obtained using a sandbox (0.1–15 kPa), pressure plate (30–100 kPa), and pressure membrane apparatus (100–1500 kPa) (Dane and Hopmans 2002). In the moderate drought and severe drought treatments, the matric suction was monitored to reach 0.8 and 1.5 MPa, respectively. After that, the plots were re-watered. In the well-watered treatment, the plots were re-watered to the field capacity by replacing the amount of water transpired every second day.

When the plant was introduced into the flower buds, drought stress was applied with field moisture curve. ABA at the early flowering stage was sprayed three times with 3 days intervals. ABA was supplied from Sigma–Aldrich Company.

### Growth and yield analyses

At the late flowering stage (85 days after planting), *D. moldavica* plant growth parameters such as plant height, number of stem branches, leaf dry weight, leaf area and total biomass were recorded. At each sampling, all *D. moldavica* plants from a 50-cm length of the three middle rows of each plot were harvested by cutting at the soil surface. The areas of green leaves were measured using a leaf area meter (model: VM-900 E/K). All plant parts were oven-dried at 70 °C for 48 h until a constant weight was reached. At maturity (about four months after planting), the plants in an area of 1 m^2^ were harvested to determine seed yield and 1000 seed weight.

### Net photosynthesis rate (PN), stomatal conductance and transpiration rate

The net photosynthesis rate, stomatal conductance and transpiration rate of leaves were measured in upper and fully opened and mature leaves using a portable infrared gas analysis (LCI, ADC BioScientific Ltd., Hoddesdon, UK). The chamber was clamped over the leaves (third from the apex) which were held horizontally in a transparent cuvette.

### Total phenolic contents

To determine total phenolic content based on Folin–Ciocalteau method, aliquots of leaf samples (0.1 g) were homogenized in deionized water (1 ml). Then 0.1 ml of the solution was mixed with 2 ml of 2% sodium carbonate (Na_2_CO_3_), 2.8 ml of deionized water, and 0.1 ml of 50% Folin–Ciocalteau reagent, and then incubated at room temperature for 30 min. Absorbance was measured at 765 nm against a deionized water blank on a spectrophotometer (Lambda 25, PerkinElmer, Waltham, MA, USA). As suggested by (Meda et al. 2005), Gallic acid was regarded as the standard.

### Essential oil yield

For extraction of essential oil (EO), 50 g dried leaves were subjected to hydro distillation using a Clevenger-type apparatus for two h (Clevenger 1928). Essential oil yield (kg ha^−1^) and percentage (%, v/ w) were estimated according to the Eqs. (1) and (2):

1 $$ {\text{Essential oil content}}\; \left( \% \right) = \frac{{{\text{mass of EO obtained }}\left( {\text{g}} \right)}}{{{\text{mass of dried leave }}\left( {\text{g}} \right)}} \times 100, $$

2 $$ {\text{Essential oil yield}} \left( {{\text{kg}} {\text{ha}}^{ - 1} } \right) = {\text{Essential}}\;{\text{oil yield}}\; \left( \% \right) \times {\text{Leaves yield }}\left( {{\text{kg}} {\text{ha}}^{ - 1} } \right). $$

### Statistical analysis

Data were subjected to an analysis of variance using PROC GLM in SAS Software (Version 9.1, SAS Institute Inc., Cary, NC), and means were compared using Duncan’s multiple range test (*P* ≤ 0.05).

## Results

### Net photosynthesis rate, stomatal conductance and transpiration rate

**Fig. 2:**
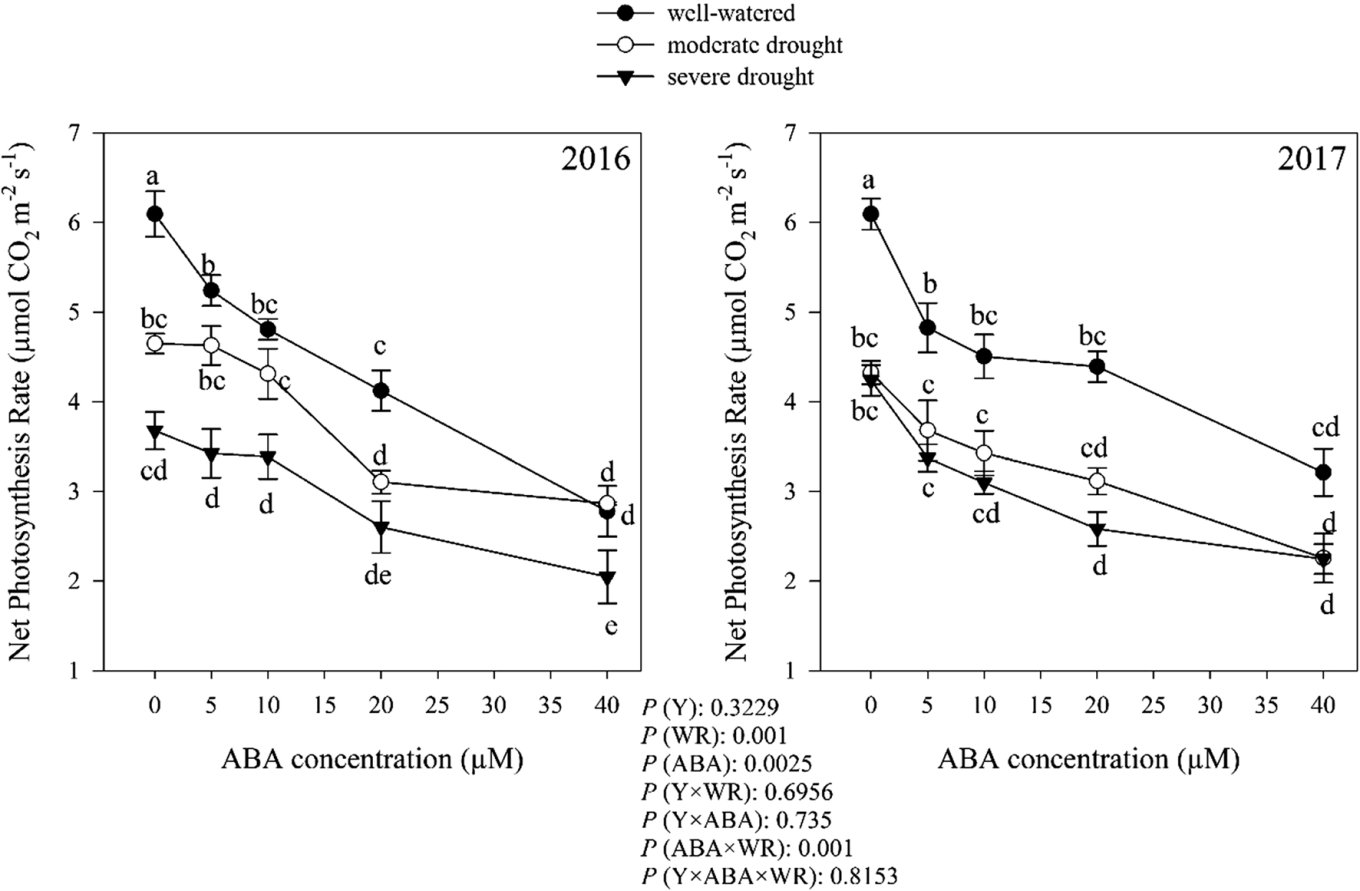
Change in net photosynthesis rate (μmol CO_2_ m^−2^ s^−1^) in dragonhead plants grown in well-watered, moderate and severe drought conditions and treated by different ABA concentrations (μM) in 2016 and 2017. Mean ± SE (*n* = 45). *P* (Y), year; *P* (WR), watering regimes effect; *P* (ABA), ABA effect; *P* (Y × WR), year × watering regimes interaction effect; *P* (Y × ABA), year × ABA interaction effect; *P* (ABA × WR), ABA × watering regimes interaction effect; *P* (Y × ABA × WR), year × ABA × watering regimes interaction effect. Different letters above the bars denote statistically significant differences between treatments at the *P* < 0.05 level according to Duncan’s test

**Fig. 3:**
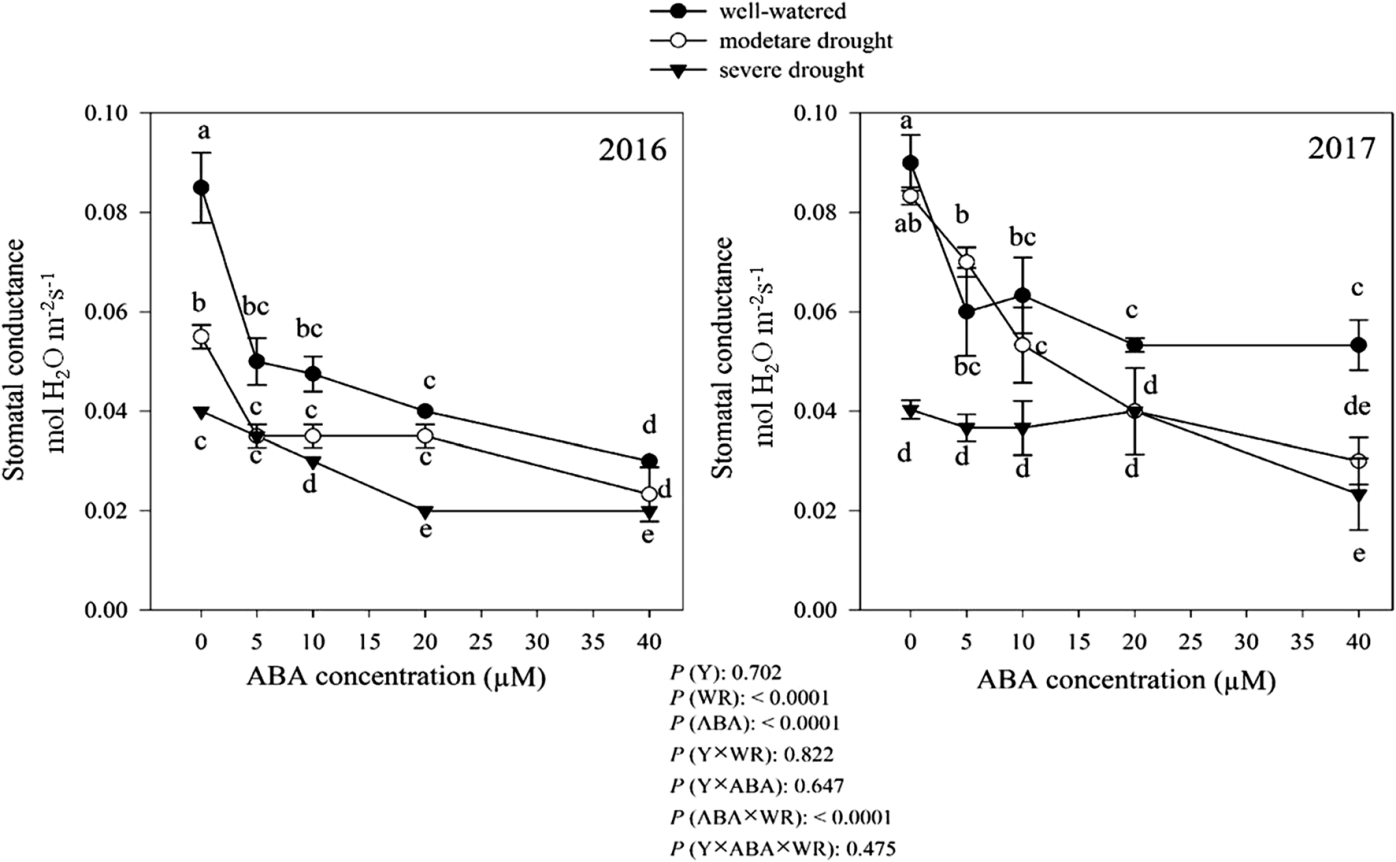
Change in stomatal conductance (mol m^−2^ s^−1^) in dragonhead plants grown in well-watered, moderate and severe drought conditions and treated by different ABA concentrations (μM) in 2016 and 2017. Mean ± SE (*n* = 45). *P* (Y), year; *P* (WR), watering regimes effect; *P* (ABA), ABA effect; *P* (Y × WR), year × watering regimes interaction effect; *P* (Y × ABA), year × ABA interaction effect; *P* (ABA × WR), ABA × watering regimes interaction effect; *P* (Y × ABA × WR), year × ABA × watering regimes interaction effect. Different letters above the bars denote statistically significant differences between treatments at the *P* < 0.05 level according to Duncan’s test

Net photosynthesis rate (PN) was similar in both years (Fig. 2). Averaged over 2 years, the highest PN was obtained from plant grown in well-watered condition, while moderate and severe drought, decreased PN by 26.39% and 34.43%, respectively (Fig. 2). The effect of ABA application and its interaction with watering regimes on PN was significant (Fig. 2). As shown in Fig. 2, there was a decreasing trend in PN associated with increasing ABA concentration in all watering regimes (Fig. 2). The response of stomatal conduction to drought and ABA application was similar to net photosynthesis rate, in this study. The results were similar in both years of the experiment (Fig. 3). Without ABA application, the highest stomatal conductance was obtained under well-watered condition, and moderate and severe drought reduced stomatal conductance by 20.97% and 54.11%, respectively (Fig. 3). Exogenous ABA application clearly reduced the stomatal conductance in all three moisture regimes (Fig. 3) and the lowest stomatal conductance (0.02 mol m^−2^ s^−1^) was obtained from plants treated by ABA 50 µM concentration under severe drought condition (Fig. 3).

**Fig. 4:**
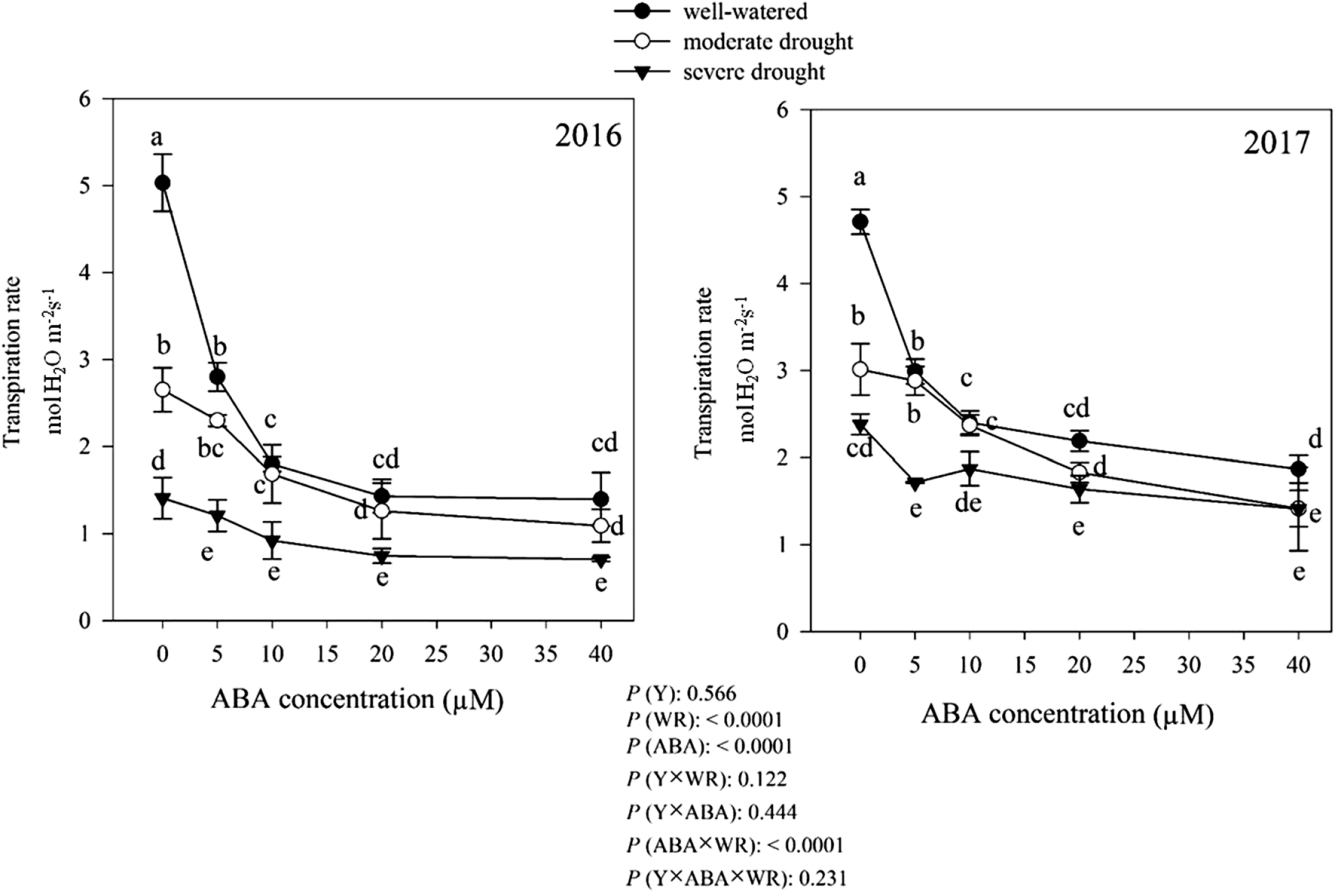
Change in transpiration rate (mmol H_2_O m^−2^ s^−1^) in dragonhead plants grown in well-watered, moderate and severe drought conditions and treated by different ABA concentrations (μM) in 2016 and 2017. Mean ± SE (*n* = 45). *P* (Y), year; *P* (WR), watering regimes effect; *P* (ABA), ABA effect; *P* (Y × WR), year × watering regimes interaction effect; *P* (Y × ABA), year × ABA interaction effect; *P* (ABA × WR), ABA × watering regimes interaction effect; *P* (Y × ABA × WR), year × ABA × watering regimes interaction effect. Different letters above the bars denote statistically significant differences between treatments at the *P* < 0.05 level according to Duncan’s test

According to the results transpiration rate of *D. moldavica* was not differ (*P* > 0.05) among two years (Fig. 4). Without ABA application, the highest transpiration rate (4.87 µmol H_2_O m^−2^ s^−1^) was obtained in well-watered condition, which was 41.82% and 61.06% more than moderate and severe drought conditions, respectively. ABA application, showed a decreasing trend in transpiration rate under well-watered and moderate drought conditions (Fig. 4). However, there was no difference between different concentrations of ABA under severe drought conditions (Fig. 4).

### Plant height

**Fig. 5:**
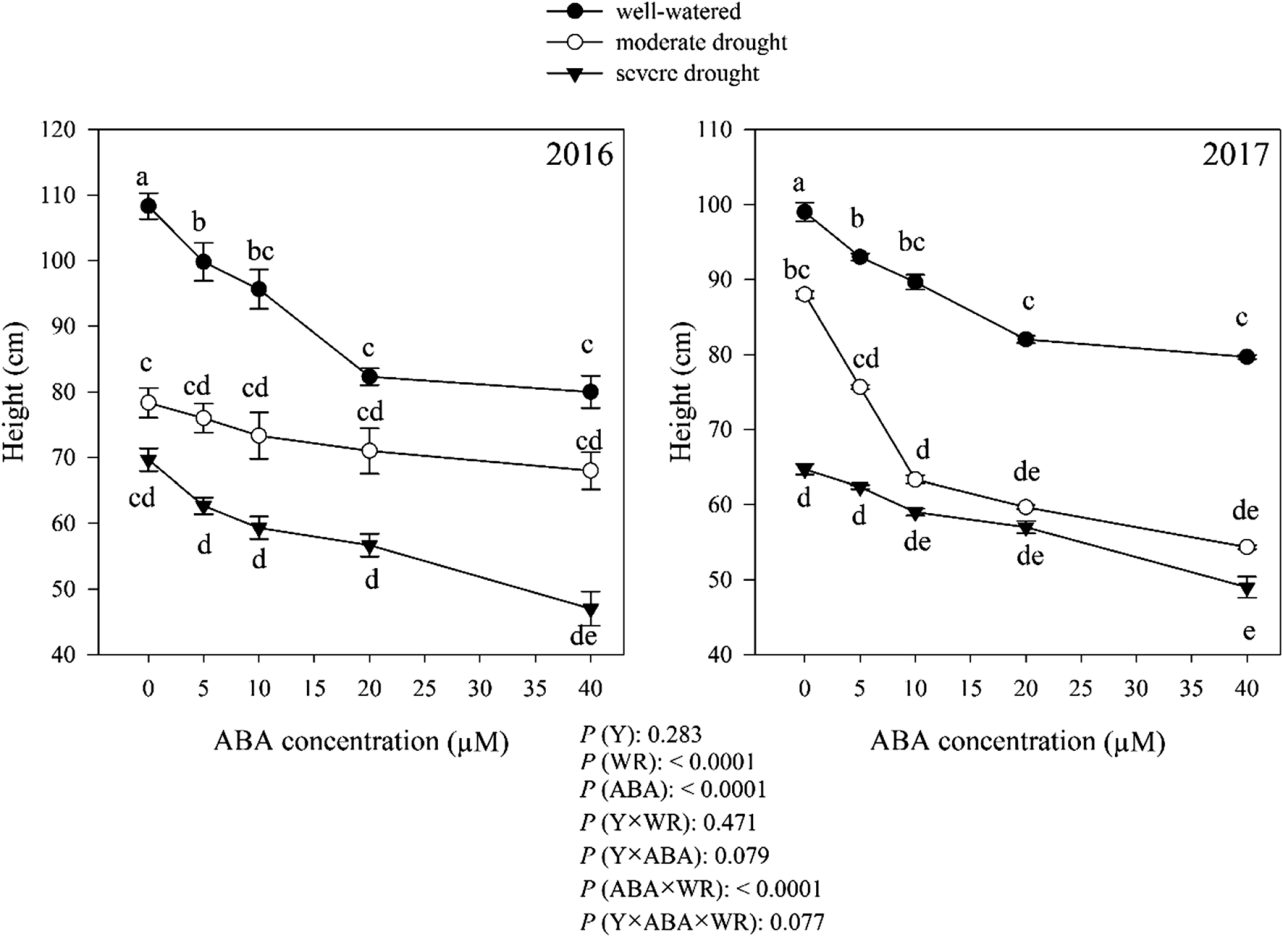
Change in plant height (cm) in dragonhead plants grown in well-watered, moderate and severe drought conditions and treated by different ABA concentrations (μM) in 2016 and 2017. Mean ± SE (*n* = 45). *P* (Y), year; *P* (WR), watering regimes effect; *P* (ABA), ABA effect; *P* (Y × WR), year × watering regimes interaction effect; *P* (Y × ABA), year × ABA interaction effect; *P* (ABA × WR), ABA × watering regimes interaction effect; *P* (Y × ABA × WR), year × ABA × watering regimes interaction effect. Different letters above the bars denote statistically significant differences between treatments at the *P* < 0.05 level according to Duncan’s test

*Dracocephalum moldavica* plant height was not different between the 2 years across treatments (*P* > 0.05). Averaged over 2 years data, the moderate and severe drought decreased plant height by 19.77 and 35.21% compared with well-watered condition. The effect of ABA and interaction of ABA × watering regimes on plant height was significant (*P* < 0.0001). Overall, plant height decreased with increasing ABA concentrations in all of moisture regimes. The lowest plant height was obtained from plants treated by ABA 40 µM in all moisture regimes, which was 23%, 26.45% and 28.54% lower than control (non-application of ABA) under well-watered, moderate and severe drought conditions, respectively (Fig. 5).

### Number of stem branch

**Fig. 6:**
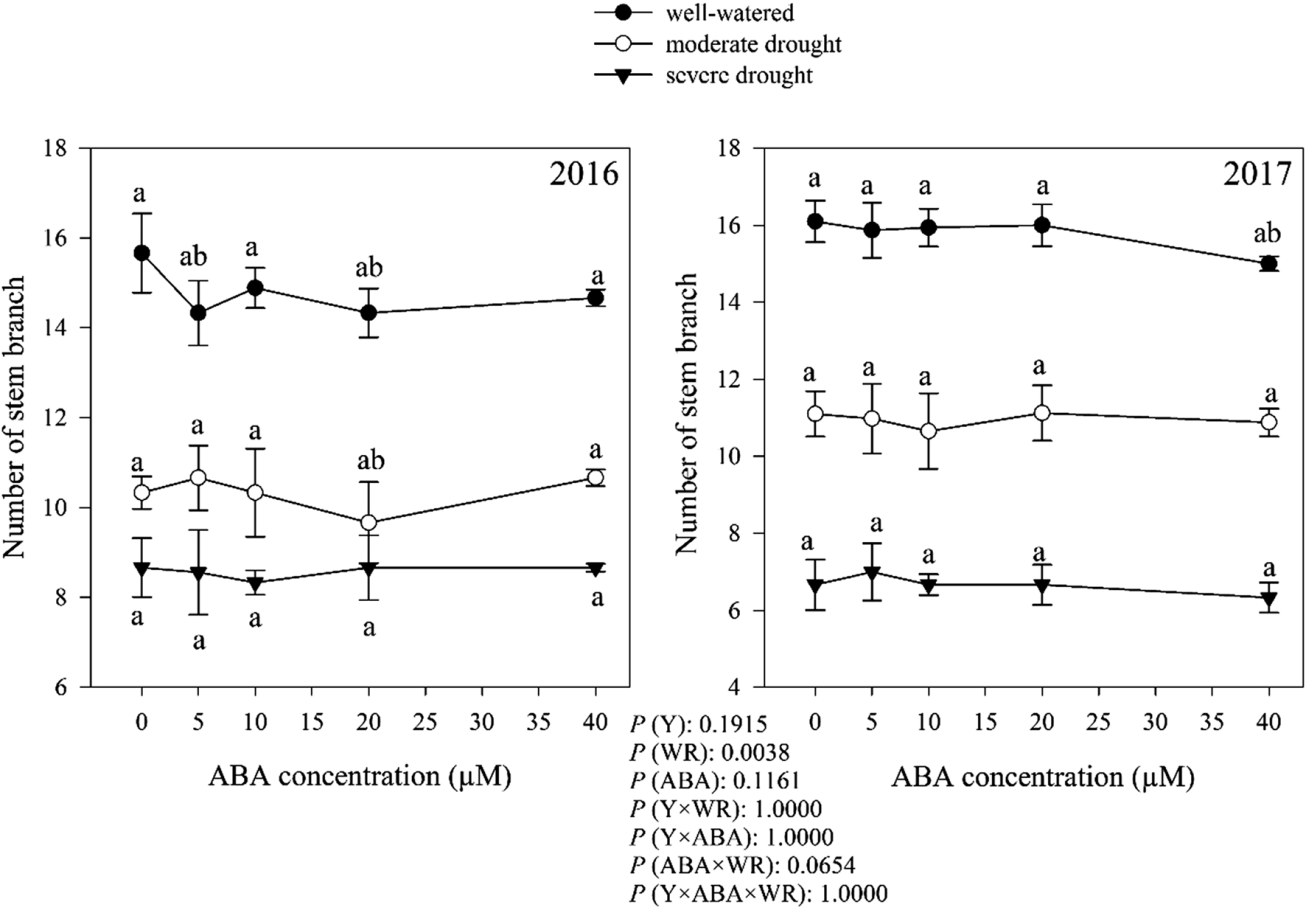
Change in number of stem branch in dragonhead plants grown in well-watered, moderate and severe drought conditions and treated by different ABA concentrations (μM) in 2016 and 2017. Mean ± SE (*n* = 45). *P* (Y), year; *P* (WR), watering regimes effect; *P* (ABA), ABA effect; *P* (Y × WR), year × watering regimes interaction effect; *P* (Y × ABA), year × ABA interaction effect; *P* (ABA × WR), ABA × watering regimes interaction effect; *P* (Y × ABA × WR), year × ABA × watering regimes interaction effect. Different letters above the bars denote statistically significant differences between treatments at the *P* < 0.05 level according to Duncan’s test

Watering regimes significantly altered the number of stem branch (*P* < 0.05). Moderate and severe drought decreased the number of stem branch 30.37 and 50.13%, respectively, compared to the well-watered condition. The effect of ABA and interaction between watering regimes and ABA on the number of stem branch was not significant, but there was a significant effect in interaction of watering regimes and ABA (Fig. 6).

### Inflorescence length

**Fig. 7:**
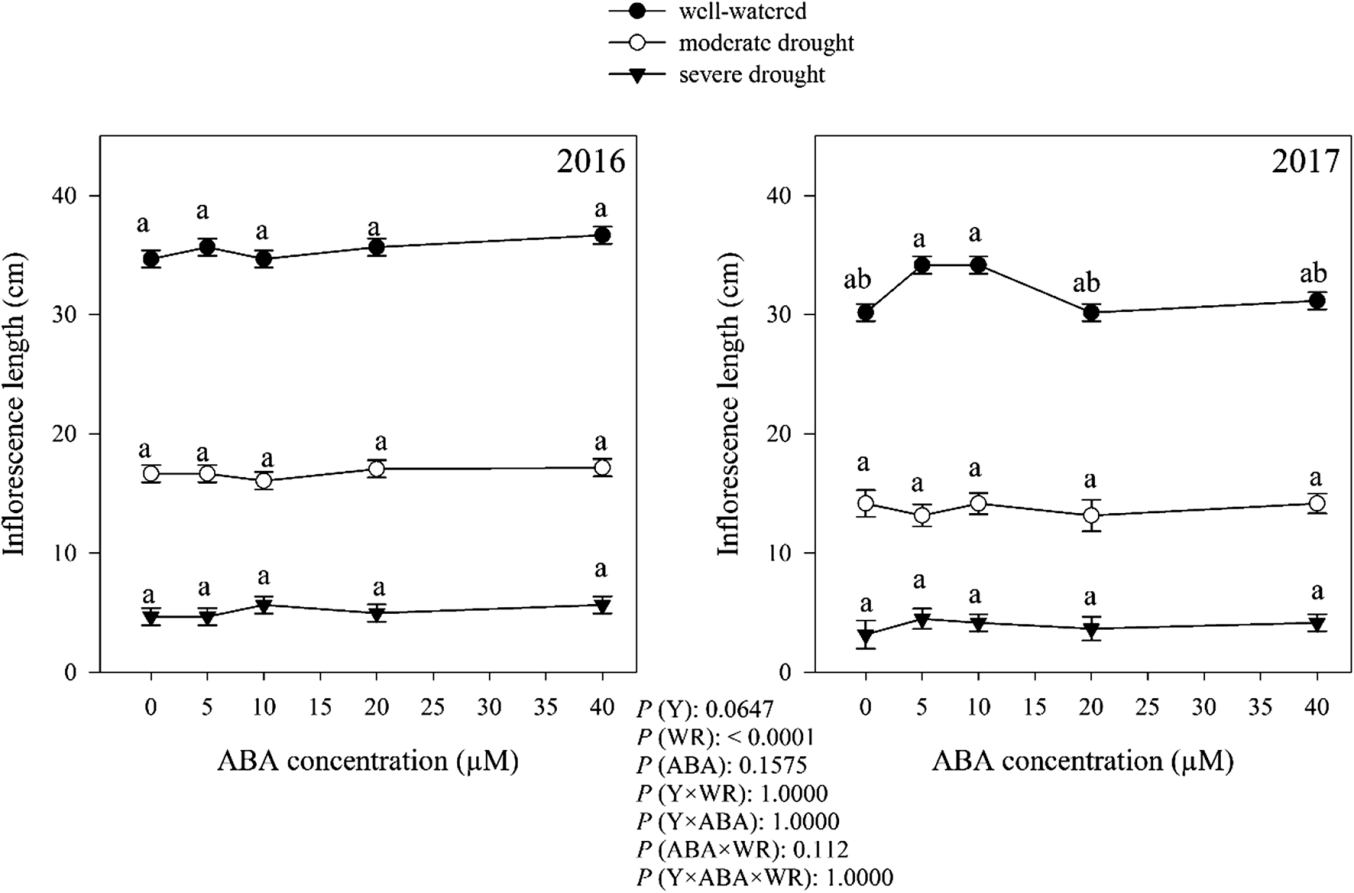
Change in Inflorescence length (cm) in dragonhead plants grown in well-watered, moderate and severe drought conditions and treated by different ABA concentrations (μM) in 2016 and 2017. Mean ± SE (*n* = 45). *P* (Y), year; *P* (WR), watering regimes effect; *P* (ABA), ABA effect; *P* (Y × WR), year × watering regimes interaction effect; *P* (Y × ABA), year × ABA interaction effect; *P* (ABA × WR), ABA × watering regimes interaction effect; *P* (Y × ABA × WR), year × ABA × watering regimes interaction effect. Different letters above the bars denote statistically significant differences between treatments at the *P* < 0.05 level according to Duncan’s test

Plant inflorescence length was not significant differ among the years. The watering regimes significantly affected plant inflorescence length but ABA concentration and interaction effects had no significant effects on this trait (Fig. 7). The highest inflorescence length (33.72 cm) was obtained from the plant grown in well-watered condition, which was 2.2 and 7.44 fold, respectively, as compared to those grown in moderate and severe drought conditions.

### Leaf area

**Fig. 8:**
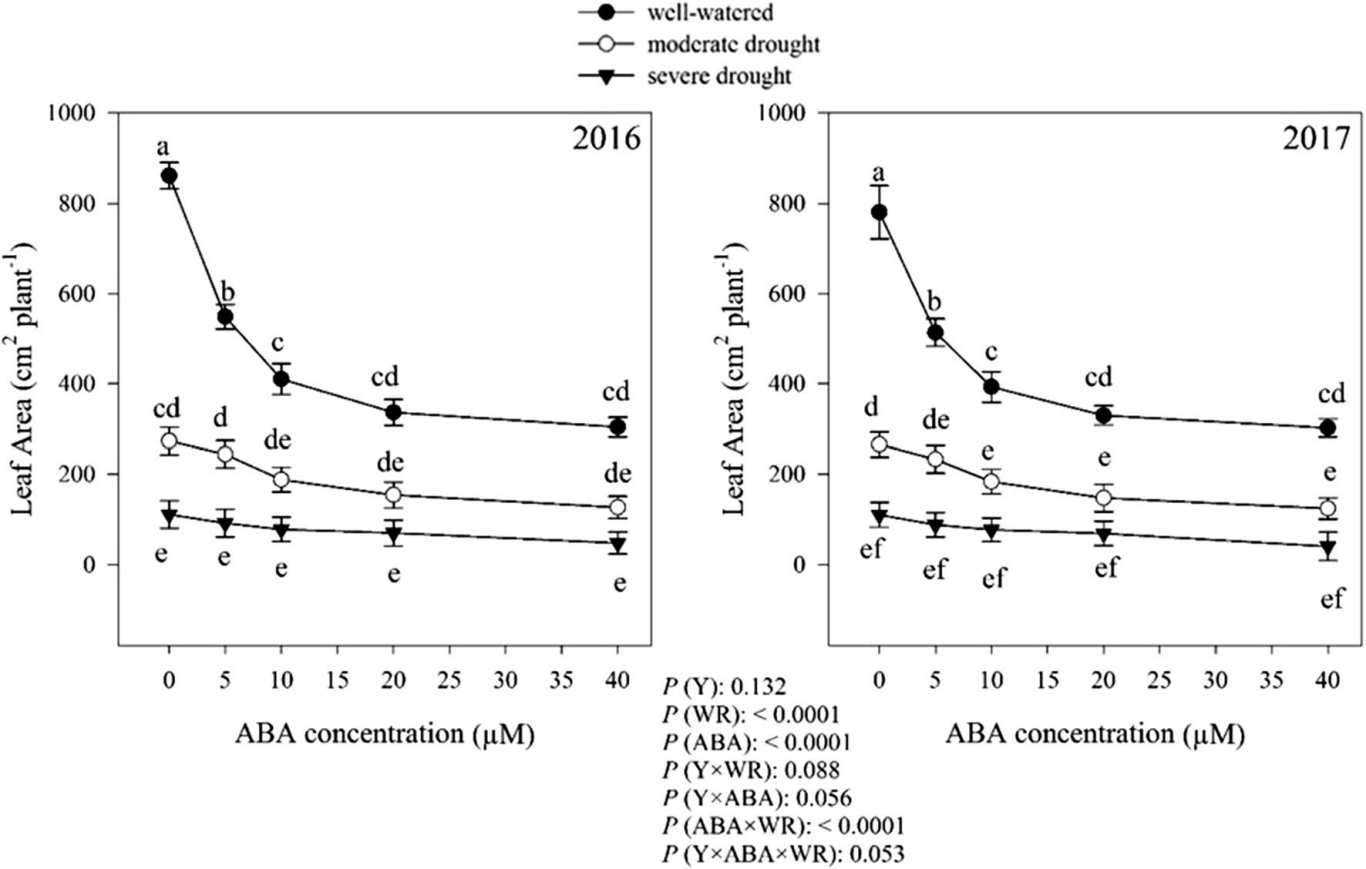
Change in leaf area (cm^2^ plant^−1^) in dragonhead plants grown in well-watered, moderate and severe drought conditions and treated by different ABA concentrations (μM) in 2016 and 2017. Mean ± SE (*n* = 45). *P* (Y), year; *P* (WR), watering regimes effect; *P* (ABA), ABA effect; *P* (Y × WR), year × watering regimes interaction effect; *P* (Y × ABA), year × ABA interaction effect; *P* (ABA × WR), ABA × watering regimes interaction effect; *P* (Y × ABA × WR), year × ABA × watering regimes interaction effect. Different letters above the bars denote statistically significant differences between treatments at the *P* < 0.05 level according to Duncan’s test

There were no significant differences (*P* > 0.05) in *D. moldavica* leaf area (LA) between years (Fig. 8). Averaged over the years, without ABA application, plants grown in the well-watered condition, had the greatest LA (820.75 cm^2^ plant^−1^), which was 67.17% and 86.53% higher than moderate and severe conditions, respectively. The effects of the variables watering regimes, ABA and interaction of ABA × watering regimes on LA were significant at *P* < 0.0001. In all three moisture regimes, leaf area showed a negative response to ABA application, and decreased with increasing ABA concentrations (Fig. 8). The lowest leaf area was obtained from 40 μM ABA concentration in all moisture regimes, which was 63.08%, 53.43% and 60.19% lower than control under well-watered, moderate and severe drought conditions, respectively.

### Leaf dry weight

**Fig. 9:**
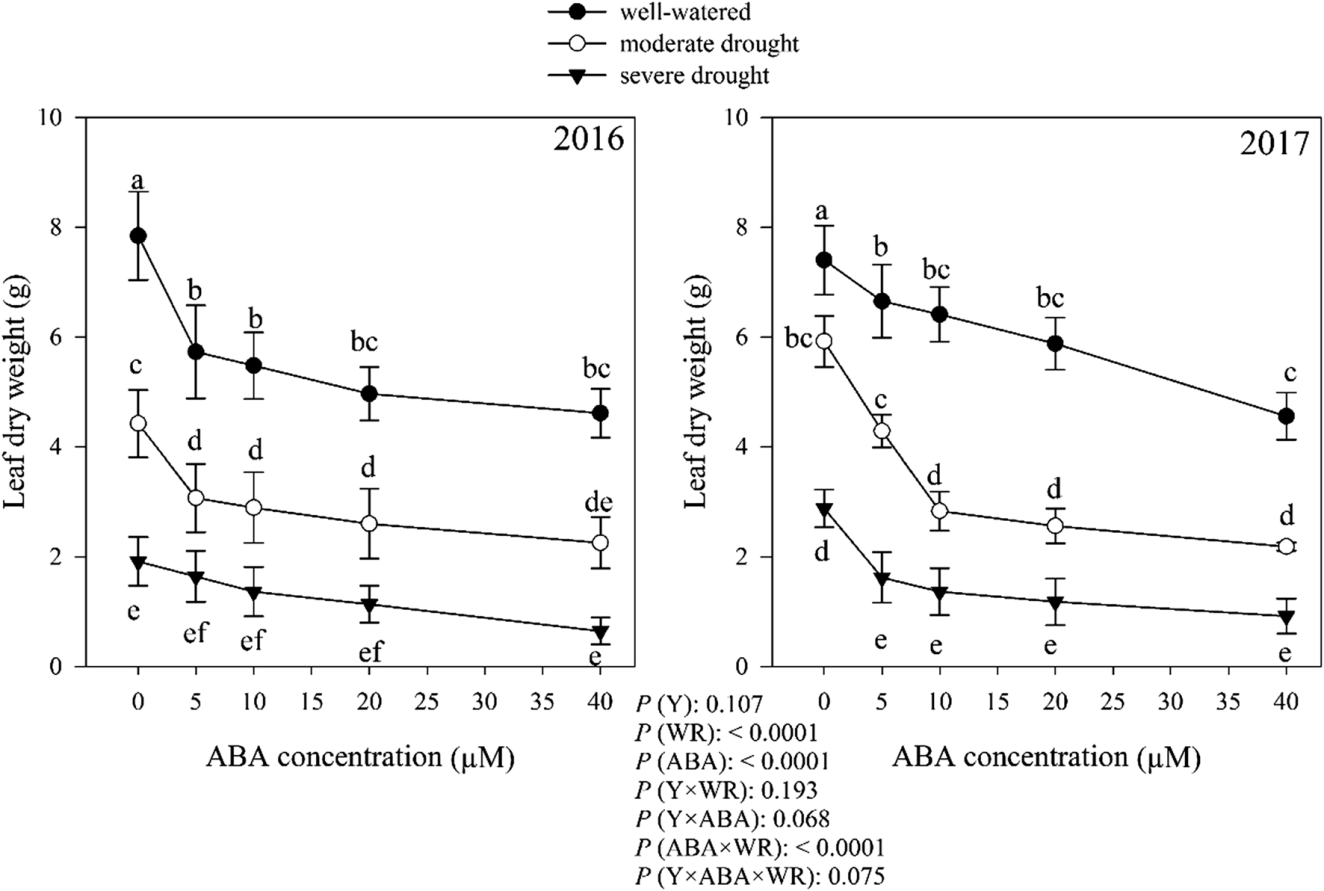
Change in leaf dry weight (g) in dragonhead plants grown in well-watered, moderate and severe drought conditions and treated by different ABA concentrations (μM) in 2016 and 2017. Mean ± SE (*n* = 45). *P* (Y), year; *P* (WR), watering regimes effect; *P* (ABA), ABA effect; *P* (Y × WR), year × watering regimes interaction effect; *P* (Y × ABA), year × ABA interaction effect; *P* (ABA × WR), ABA × watering regimes interaction effect; *P* (Y × ABA × WR), year × ABA × watering regimes interaction effect. Different letters above the bars denote statistically significant differences between treatments at the *P* < 0.05 level according to Duncan’s test

According to the results of variance analysis, leaf dry weight (LDW) did not differ (*P* > 0.05) among 2 years. Without ABA application, moderate and severe drought caused 18.05% and 30.15% reduction in LDW compared with well-watered condition, respectively. Watering regimes, ABA and interaction of ABA × watering regimes effects on LDW were significant at *P* < 0.0001. With increasing ABA concentration, LDW decreased, and the highest and lowest LDW was obtained from control and ABA 40 μM concentration, respectively, in three moisture conditions (Fig. 9).

### Biological yield

**Fig. 10:**
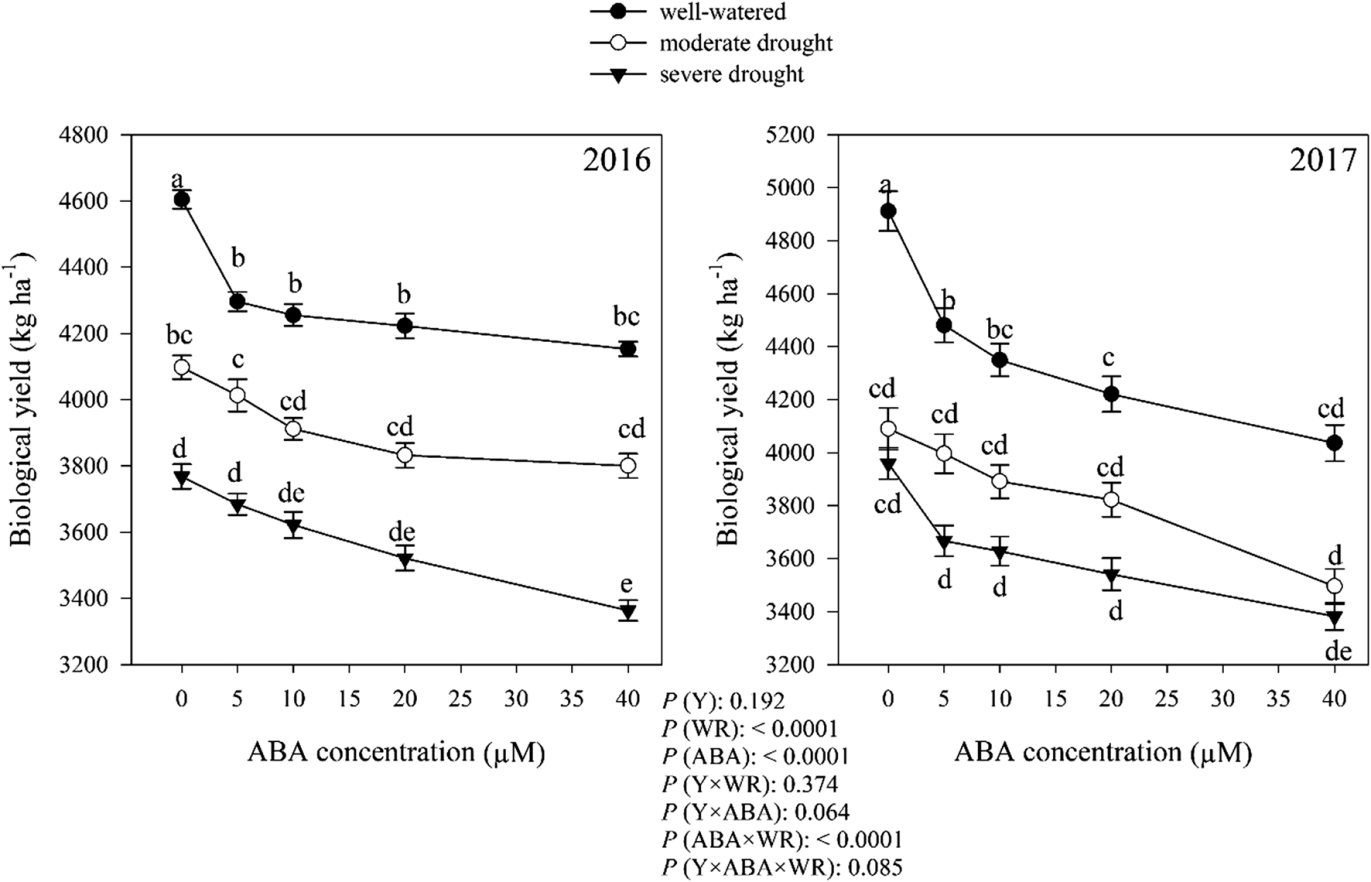
Change in biological yield (kg ha^−1^) in dragonhead plants grown in well-watered, moderate and severe drought conditions and treated by different ABA concentrations (μM) in 2016 and 2017. Mean ± SE (*n* = 45). *P* (Y), year; *P* (WR), watering regimes effect; *P* (ABA), ABA effect; *P* (Y × WR), year × watering regimes interaction effect; *P* (Y × ABA), year × ABA interaction effect; *P* (ABA × WR), ABA × watering regimes interaction effect; *P* (Y × ABA × WR), year × ABA × watering regimes interaction effect. Different letters above the bars denote statistically significant differences between treatments at the *P* < 0.05 level according to Duncan’s test

Watering regimes, ABA and their interaction significantly (*P* < 0.0001) altered plant biological yield, but the effect of the year treatment was not significant (*P* > 0.05) (Fig. 10). Without ABA application, moderate and severe drought decreased biological yield by 18.17% and 39.83%, respectively, compared to well-watered condition. ABA application caused a descending trend in biological yield, meaning that biological yield decreased with increasing ABA concentration in all moisture conditions (Fig. 10).

### Seed yield

**Fig. 11:**
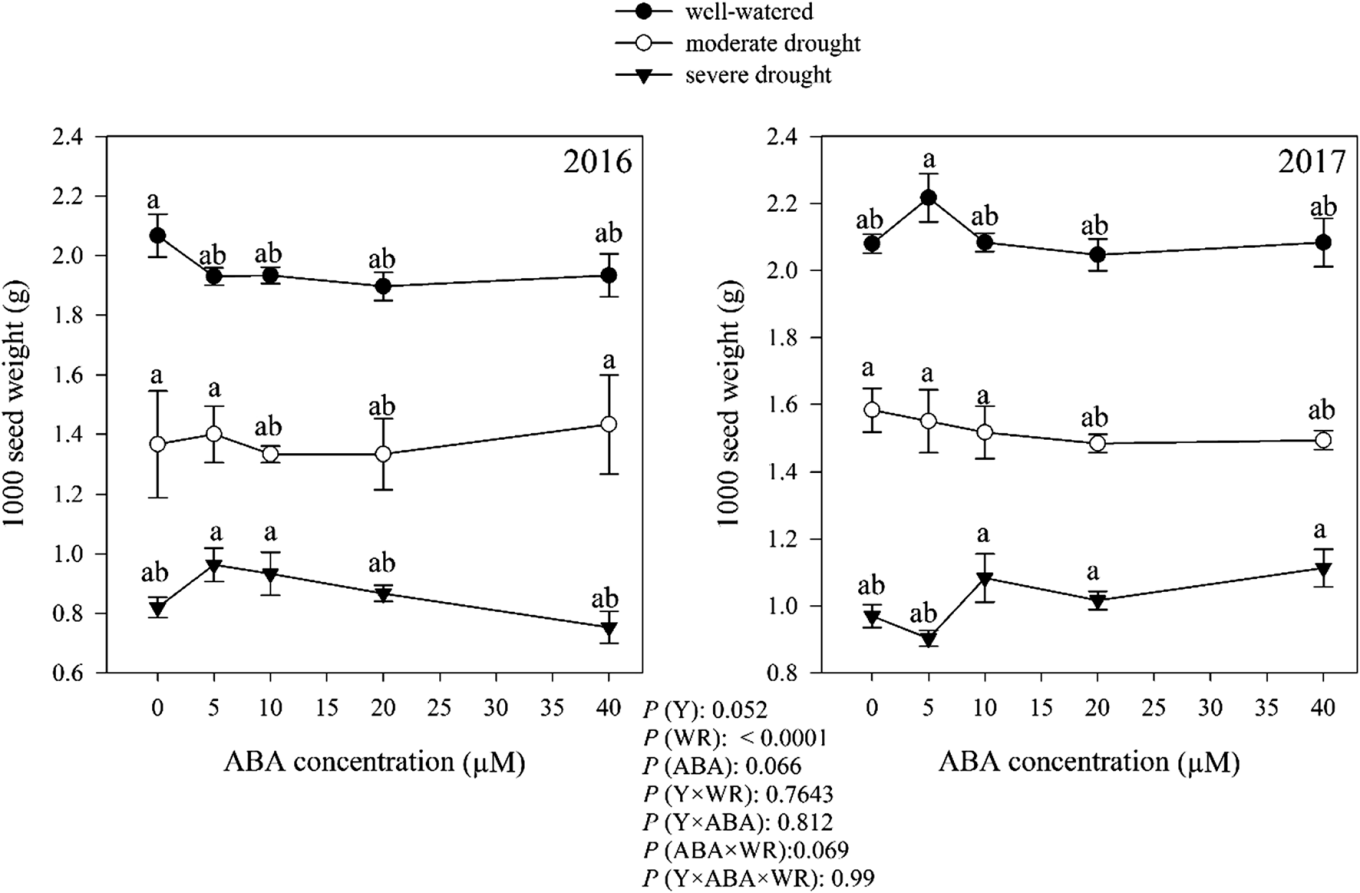
Change in 1000 seed weight (*g*) in dragonhead plants grown in well-watered, moderate and severe drought conditions and treated by different ABA concentrations (μM) in 2016 and 2017. Mean ± SE (*n* = 45). *P* (Y), year; *P* (WR), watering regimes effect; *P* (ABA), ABA effect; *P* (Y × WR), year × watering regimes interaction effect; *P* (Y × ABA), year × ABA interaction effect; *P* (ABA × WR), ABA × watering regimes interaction effect; *P* (Y × ABA × WR), year × ABA × watering regimes interaction effect. Different letters above the bars denote statistically significant differences between treatments at the *P* < 0.05 level according to Duncan’s test

1000-seed weight was only significantly affected by the effect of watering regimes (Fig. 11). Averaged over the years, the highest 1000-seed weight (2.03 g) was obtained from well-watered condition (Fig. 11), which was 28.57% and 53.69% higher than moderate and severe drought conditions, respectively.

**Fig. 12:**
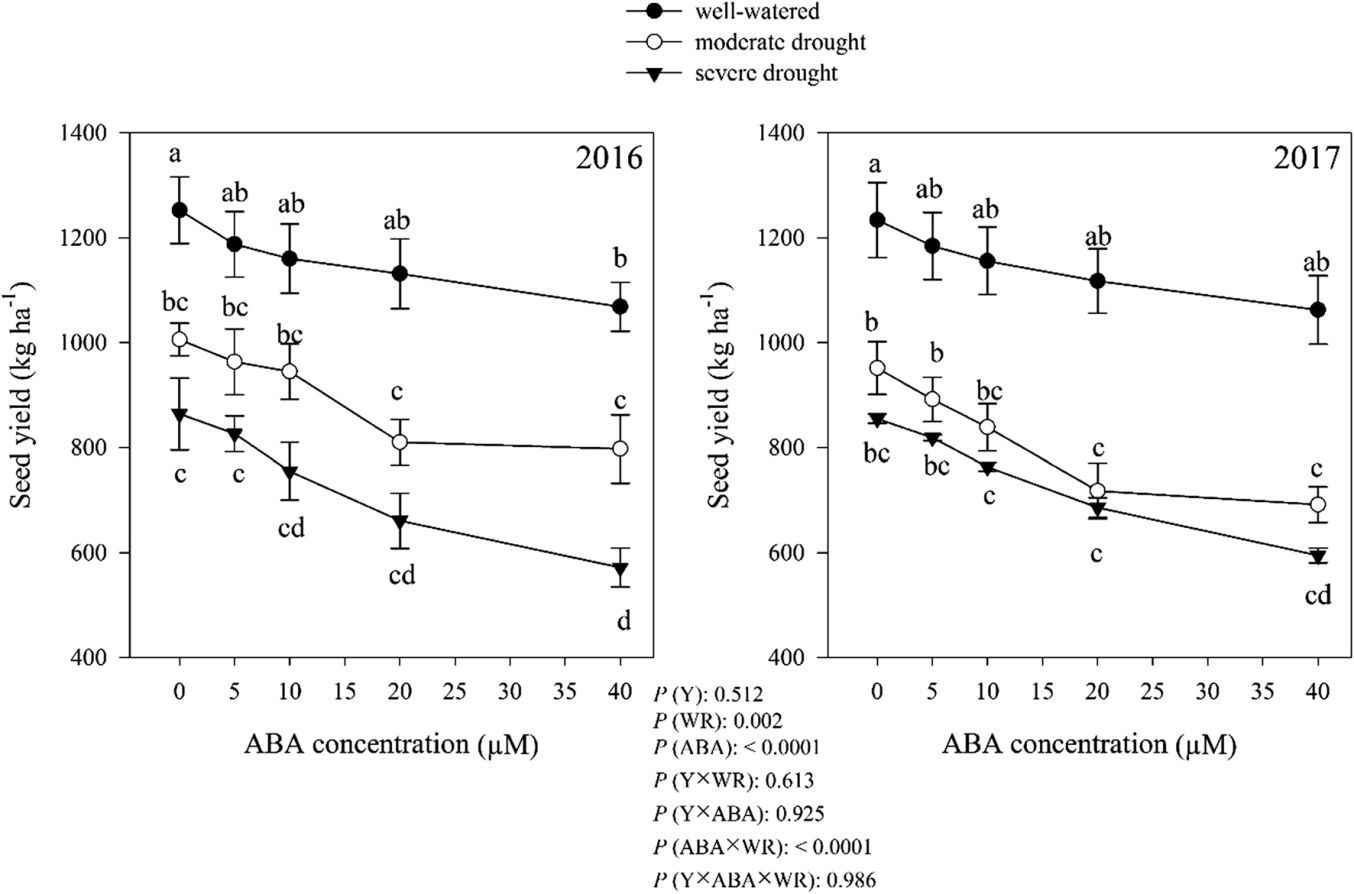
Change in seed yield (kg ha^−1^) in dragonhead plants grown in well-watered, moderate and severe drought conditions and treated by different ABA concentrations (μM) in 2016 and 2017. Mean ± SE (*n* = 45). *P* (Y), year; *P* (WR), watering regimes effect; *P* (ABA), ABA effect; *P* (Y × WR), year × watering regimes interaction effect; *P* (Y × ABA), year × ABA interaction effect; *P* (ABA × WR), ABA × watering regimes interaction effect; *P* (Y × ABA × WR), year × ABA × watering regimes interaction effect. Different letters above the bars denote statistically significant differences between treatments at the *P* < 0.05 level according to Duncan’s test

*Dracocephalum moldavica* seed yield was significantly affected by watering regimes, ABA and interactions among the independent variable. The experiment results were not different for 2 consecutive years (*P* > 0.05). Averaged over the 2 years, without ABA application, moderate and severe drought caused 21.21% and 30.88% reduction in seed yield compared with well-watered condition, respectively. As shown in Fig. 12, increase in ABA concentrations was associated with reduction of seed yield. The lowest seed yield was obtained from plants treated with 40 μM ABA in all three moisture regimes (Fig. 12), which was less than control under well-watered, moderate and severe drought conditions 14.28%, 23.94% and 32.13%, respectively.

### Total phenolic content

**Fig. 13:**
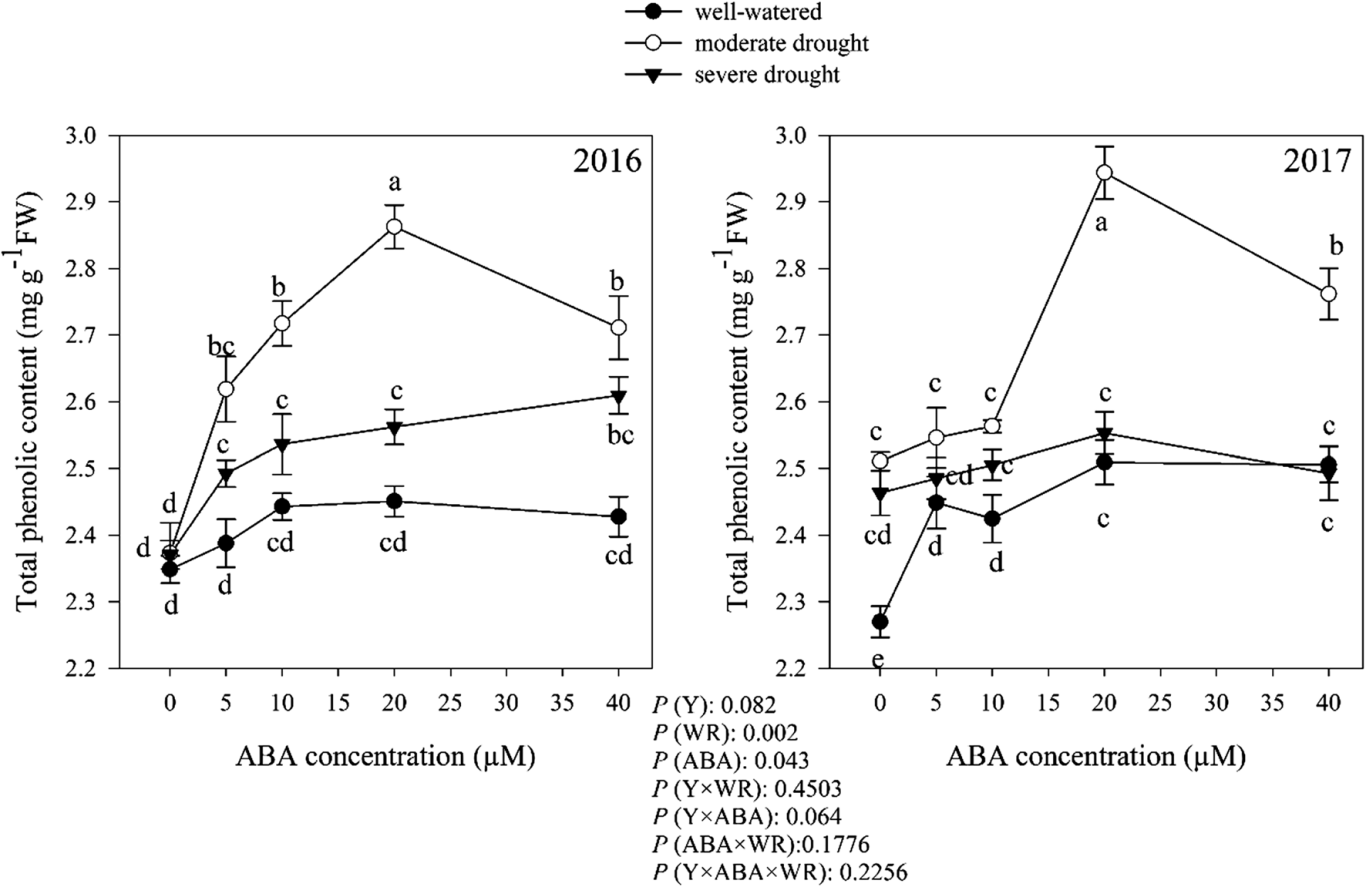
Change in total phenolic content (mg g^−1^ FW) in dragonhead plants grown in well-watered, moderate and severe drought conditions and treated by different ABA concentrations (μM) in 2016 and 2017. Mean ± SE (*n* = 45). *P* (Y), year; *P* (WR), watering regimes effect; *P* (ABA), ABA effect; *P* (Y × WR), year × watering regimes interaction effect; *P* (Y × ABA), year × ABA interaction effect; *P* (ABA × WR), ABA × watering regimes interaction effect; *P* (Y × ABA × WR), year × ABA × watering regimes interaction effect. Different letters above the bars denote statistically significant differences between treatments at the *P* < 0.05 level according to Duncan’s test

As shown in Fig. 13, total phenolic content were affected by watering regimes, ABA and interaction among the independent variables. Total phenolic content was not different (*P* > 0.05) among 2 years (Fig. 13). Without the ABA treatment, the highest total phenolic content was obtained from plants grown in moderate drought conditions (Fig. 13), respectively, 5.64 and 9.02% higher than plants grown in severe drought conditions and in good irrigation conditions. However, depending on watering regimes, plants obviously showed a different response to ABA treatment in terms of total phenolic content. Overall, the maximum total phenolic content (8.9 mg g^−1^FW) was obtained in moderate drought condition with the application of 20 μM ABA while the future increase in ABA concentration decreased it (Fig. 13). There was an increasing trend in total phenolic content with increasing in ABA concentration up to 20 μM in the plant grown well-watered and severe drought conditions and then it remained almost stable up to 40 μM ABA (Fig. 13).

### Essential oil content and yield

**Fig. 14:**
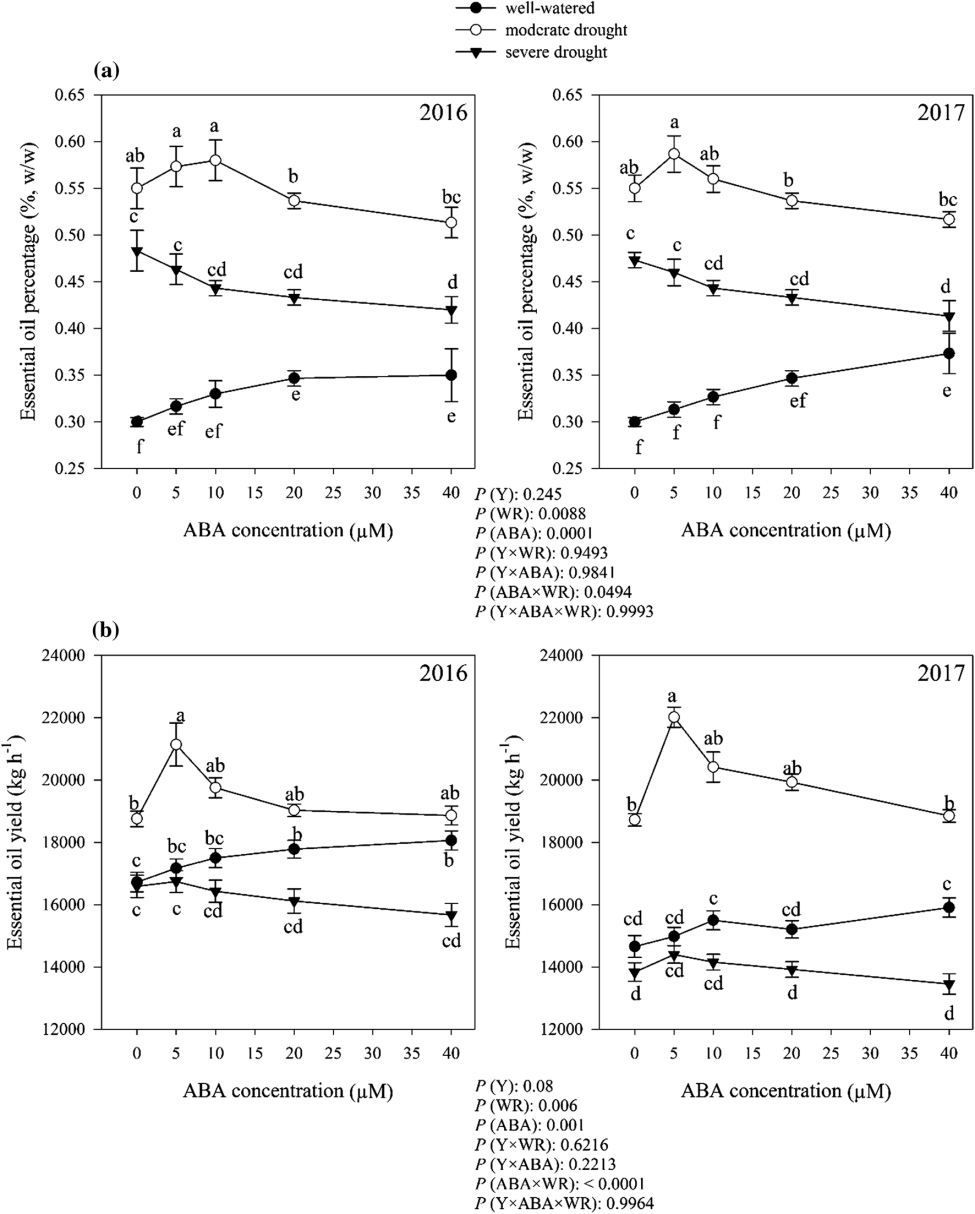
Change in essential oil percentage (%, w/w) **a** and essential oil yield (kg ha^−1^) **b** in dragonhead plants grown in well-watered, moderate and severe drought conditions and treated by different ABA concentrations (μM) in 2016 and 2017. Mean ± SE (*n* = 45). *P* (Y), year; *P* (WR), watering regimes effect; *P* (ABA), ABA effect; *P* (Y × WR), year × watering regimes interaction effect; *P* (Y × ABA), year × ABA interaction effect; *P* (ABA × WR), ABA × watering regimes interaction effect; *P* (Y × ABA × WR), year × ABA × watering regimes interaction effect. Different letters above the bars denote statistically significant differences between treatments at the *P* < 0.05 level according to Duncan’s test

Essential oil content of *D. moldavica* significantly affect by watering regimes, ABA application and interactions between treatments (*P* < 0.01). Averaged over 2 years data, without ABA application, *D. moldavica* plants grown under moderate and severe drought conditions, respectively, had 45.45% and 37.5% more essential oil content, than well-watered conditions. The maximum essential oil content (0.58%) accumulated in plants that grown under moderate drought condition and treated with 5 µM ABA (Fig. 14a).

The essential oil yield was significantly affected by watering regimes, ABA and interactions among the independent variable (Fig. 14b). Without ABA application, plant grown under moderate drought had higher essential oil yield than plants grown under well-watered and severe drought conditions (Fig. 14b). Plant essential oil yield response to ABA application varied depending on the moisture conditions (Fig. 14b). In the well-watered condition, application of ABA had a positive effect on essential oil yield and there was an increasing trend in essential oil yield, associated with increasing ABA concentration (Fig. 14b). The highest essential oil yield (19.58 kg ha^−1^) was obtained from 5 µM ABA concentration under moderate drought condition (Fig. 14b). In the severe condition, essential oil yield was not significantly affected by the ABA exogenous application (Fig. 14b).

## Discussion

In this study, it was evaluated how different water stress conditions can influence many physiological parameters of *D. moldavica*. Furthermore, although the endogenous signaling of ABA is known to improve the response of plants to drought, *D. moldavica* exposed to exogenous ABA showed heterogeneous responses.

Compared to well-watered condition, drought stress severely decreased PN in *D. moldavica* plants (Fig. 2). Stomatal closure (shown in Fig. 3) due to drought leads to a decrease in intercellular CO_2_ concentrations (data not shown). In addition, drought can disturbs the photosynthesis pathway key enzymes activity such as ribulose–1, 5–bisphosphate carboxylase–oxygenase (Rubisco) and fructose–1,6–bisphosphatase (FBP) (Webber et al. 1994; Vu et al. 1999). According to our results, PN decreased with increasing in ABA concentration (Fig. 2). Our results indicated that a reduction in PN with increasing ABA concentration (Fig. 2) attributed to diffusive related to stomatal conductance limitations in ABA-treated plants (shown in Fig. 3). ABA can regulate stomatal closure by Ca^2+^ and K^+^ outflowing in the guard cell membrane (Pei et al. 2000) and causing photosynthesis inhibition (Zhou et al. 2006).

In this study, drought stress markedly reduced leaf area, leaf dry weight, height, biological yield, seed yield of *D. moldavica* plants compared to well-watered plants, and this effect was more pronounced with the severity of drought (Fig. 8). Under drought conditions, leaf area was sharply reduced due to reduction in leaf growth and increasing leaf abscission (Anyia and Herzog 2004).

The reduction in photosynthetic yield caused by ABA is closely associated with the development of photosynthetic organs such as leaves. Indeed, leaf area responded negatively to increasing concentration of ABA. ABA plays a key role in stomatal regulation (Wu et al. 1997). The causal association between elevated ABA levels and reduced stomatal aperture under stress conditions has been well established (Hsiao et al. 1976). The application of exogenous ABA causes reduction in the stomatal conductance and hence PN (Li et al. 2004), which subsequently leads to a decrease in growth and number and area of the leaves due to the decrease in assimilates flow. The leaf production in ABA-treated watermelon (Agehara and Leskovar 2014) and *Catharanthus* plants (Jaleel et al. 2006) was reduced. In addition, ABA reduced the leaf number in *Ocimum sanctum* (Nair et al. 2009). The plant height also decreased under stressful conditions. In drought stress conditions because of reduction in citokinins transport from root to shoot or because of an increased amount of ABA in leaves, the flexibility of the cells wall decreased, so plant growth is reduced. El-antably (1974) reported that ABA reduced plant height of corn and sorghum significantly by about 15 and 11%, respectively, by cessation of extension growth in both corn and sorghum plants. This may be an indirect effect of ABA on inhibition of shoot growth. The application of ABA reduced plant height. ABA can act as an inhibitor of the elongation of the stem in some plants, including eggplant (Latimer and Mitchell 1988), cucumber (Yamazaki et al. 1995) and pepper (Biai et al. 2011).

The reduction of the dry weight of the leaves of the plant and the biological yield of the *D. moldavica* are shown in Figs. 9 and 10. Leaf dry weight and biomass accumulation are very sensitive to water deficit because they are dependent on cell expansion (Hsiao et al. 1976; Hearn 1994). Loka et al. (2011) reported that drought caused a reduction in the whole plant leaf area by decreasing the initiation of new leaves and a decrease in leaf size. The result of reduced leaf growth is the reduction of biomass accumulation. A common detrimental effect of water deficit on plants production is the reduction in fresh and dry biomass production (Zhao et al. 2006). Kamara et al. (2003) reported that drought significantly reduced total dry weight in maize. Leaf dry weight and biological yield decreased by application of ABA (Figs. 9 and 10). ABA has also been shown to be involved in mobilization of reserves under drought stress conditions. Yin et al. (2004) reported that exogenous application of ABA in *Populus kangdingensis* and *Populus cathayana* reduced total dry weight. ABA is also involved in many physiological processes, such as photosynthesis. It has been demonstrated that ABA plays important roles in stomatal conductance (Phillips et al. 1997), the stability of photosynthetic apparatus (Xu et al. 1995; Gong et al. 1998) and the assimilates production (Alamillo and Bartels 2001). Plant production and finally biological yield of *D. moldavica* plant decreased due to reduced photosynthetic activity.

The seed yield decreased under drought stress conditions in this experiment. Under water deficit, the duration of seed filling may be controlled by the increased rate of leaf senescence, which regulated by the nitrogen status of the plant (De Souza et al. 1997). Drought stress during seed filling generally decreases nitrogen accumulation of new plant tissues (Frederick and Camberato 1995). Drought mainly influences seed yield by limiting seed numbers by either influencing the amount of dry matter produced by the time of flowering or by directly influencing pollen or ovule function, which leads to decreased seed-set. Secondarily, drought influences seed filling mainly by limiting the assimilate supply, leading to smaller seed size and lower yields (Frederick et al. 1991).

ABA application decreased seed yield (Fig. 12). Studies with exogenous application of ABA suggest that cell division and developing processes under full water conditions, showed similar responses to those of water deficit and led to lower seed-set and seed development (Myers et al. 1990; Mambelli and Setter 1998). Trivedi et al. (2018) reported that ABA application reduced seed yield in maize.

Interesting data were observed on the variation of the total phenol content under water stress conditions and at increasing concentrations of ABA. Total phenolic content increased under drought conditions (Fig. 13). Exposure to some environmental stress, such as drought often increases the production of reactive oxygen species (ROS) (Sánchez-Rodríguez et al. 2011). ROS can produce antioxidants and secondary metabolism such as phenolic compounds which play an important role in the detoxification of ROS (Ksouri et al. 2007). Shikimate is the main phenolic compounds biosynthesis pathway (Parida et al. 2004) and phenylalanine ammonium lyase (PAL, EC 4.3.1.5) is a key enzyme. Some data reports that the PAL activity increases in drought conditions by increasing the plant phenolic compounds (Keleş and Öncel 2002; Oh et al. 2009). Moreover, exogenous ABA can induce an increase in the biosynthesis of flavonoids and carotenoids in conditions of water stress (Gai et al. 2020). Indeed, phenolic content improved with increasing in ABA concentration in well-watered and severe drought conditions (Fig. 13). In addition, ABA increased total phenolic content up to 20 µM concentration in moderate drought (Fig. 13). Shen et al. (2016) reported that ABA could improve the expressions of PAL and tyrosine aminotransferase (TAT) genes that are key enzymes in the phenolic biosynthesis pathway. From these data, it can be assumed that the increase in phenolic compounds can be induced up to certain levels of water stress in *D. moldavica*. Indeed, severe drought decreases the production of these secondary metabolites in both years. Doses higher than 20 µM of exogenous ABA are no longer efficient in increasing phenolic compounds in conditions of moderate drought in *D. moldavica*. We can hypothesize that to counteract plant stress, exogenous ABA induces an enzymatic mechanism for the synthesis of new phenolic compounds which is regulated by the increase in ABA levels. When the exogenous ABA exceeds the concentration of 20 µM it has an inhibitory effect (negative feedback) on the action of the enzyme (or even on the expression of the enzyme itself).

Essential oil content significantly affected by drought (Fig. 14a, b). Akula and Ravishankar (2011) have reported that plants increased essential oil content as a defense system to protect against ROS when exposed to drought conditions. Selmar and Kleinwächter (2013) showed that the total monoterpenes concentration of sage plant was significantly higher under drought stress compared to normal irrigated plants. The results of the present study showed that, under severe drought conditions, the essential oil yield decreased despite the increasing in essential oil percentage, (Fig. 14a, b). In drought conditions, accumulation of plants biomass decreases, so the percentage of essential oil increases without increasing in the biosynthesis of the essential oil content. However, drought increases the activity of the essential oil biosynthetic pathway enzymes (Selmar et al. 2017). Since the essential oil yield is related to the interaction between the essential oil percentage and dry weight production (Baher et al. 2002), increasing drought intensity leads to an increase in the essential oil percentage and decreases leaf yield, which ultimately reduces the essential oil yield.

ABA application affected the percentage and yield of essential oil. The highest percentage and yield of essential oil obtained in moderate drought condition with the application of 5 μM ABA (Fig. 14a and b). Terpenoids are the main components of essential oil. 1–deoxy–D–xylulose 5–phosphate synthase (DXS) and 3–hydroxy–3–methylglutaryl–CoA reductase (HMGR) are two of the key enzymes in the pathway of biosynthesis of the terpenoids (Lichtenthaler 1999). ABA can increase DXS and HMGR activity in treated plants (Mansouri and Asrar 2012; Jacobo-Velázquez et al. 2015; Wang et al. 2015).

It is interesting to note that in conditions of moderate drought the maximum effect of exogenous ABA on the increase of essential oils is reached at 5 μM compared to 20 µM for the synthesis of phenolic compounds. This dual response suggests that different dosages are required for the activation of the two biosynthetic pathways by exogenous ABA in *D. moldavica*.

## Conclusion

In this study, we analyzed the roles of exogenous ABA on some physiological, morphological and metabolic parameters of *D. moldavica* under drought stress. The study showed that severe and moderate drought reduce photosynthetic efficiency and alter transpiation processes and leaf morphology. Exogenous ABA does not restore these impaired functions but improves secondary metabolism. *D. moldavica* produces high quantities of some secondary metabolites such as phenolic compounds and essential oil as a response to stress condition. In particular, a concentration of 5 µM of exogenous ABA is required to activate the biosynthetic pathway of essential oils and 20 µM for phenolic compounds in *D. moldavica* under moderate drought. This can be explained by the existence of multiple biosynthetic pathways of the secondary metabolites of *D. moldavica* activated by different dosages of ABA. Unfortunately, a critical aspect of this manuscript is that it did not assess endogenous ABA levels. The quantification of the endogenous levels of ABA could have better clarify the signaling involved in the biosynthetic pathways of phenols and essential oils. Therefore, extreme ecological conditions and treatment with ABA should be considered economically and pharmaceutically significant in *D. moldavica* cultivation, especially in arid and semi-arid regions. This study will contribute to the understanding of the abiotic stress resilience mechanism in *D. moldavica* and provide new insights to improve *D. moldavica* drought tolerance in the future.

## Data Availability

The data will be made available on reasonable request.

## Abbreviations

ABA: Abscisic acid
PN: Net photosynthesis rate
ROS: Reactive oxygen species
